# Supplementary material for: Molecular anatomy of PLK1 master docking motifs
Source: Nat Commun. 2026 May 11;17:4228. doi: 10.1038/s41467-026-73038-8 (PMC13161223; doi:10.1038/s41467-026-73038-8)
Supplement: Supplementary file 1 — Supplementary Information [file 41467_2026_73038_MOESM1_ESM.pdf]

Molecular anatomy of PLK1 master docking motifs

by Long Ren et al.

Supplementary Information

including

Supplementary Figures S1 to S11 and their legends

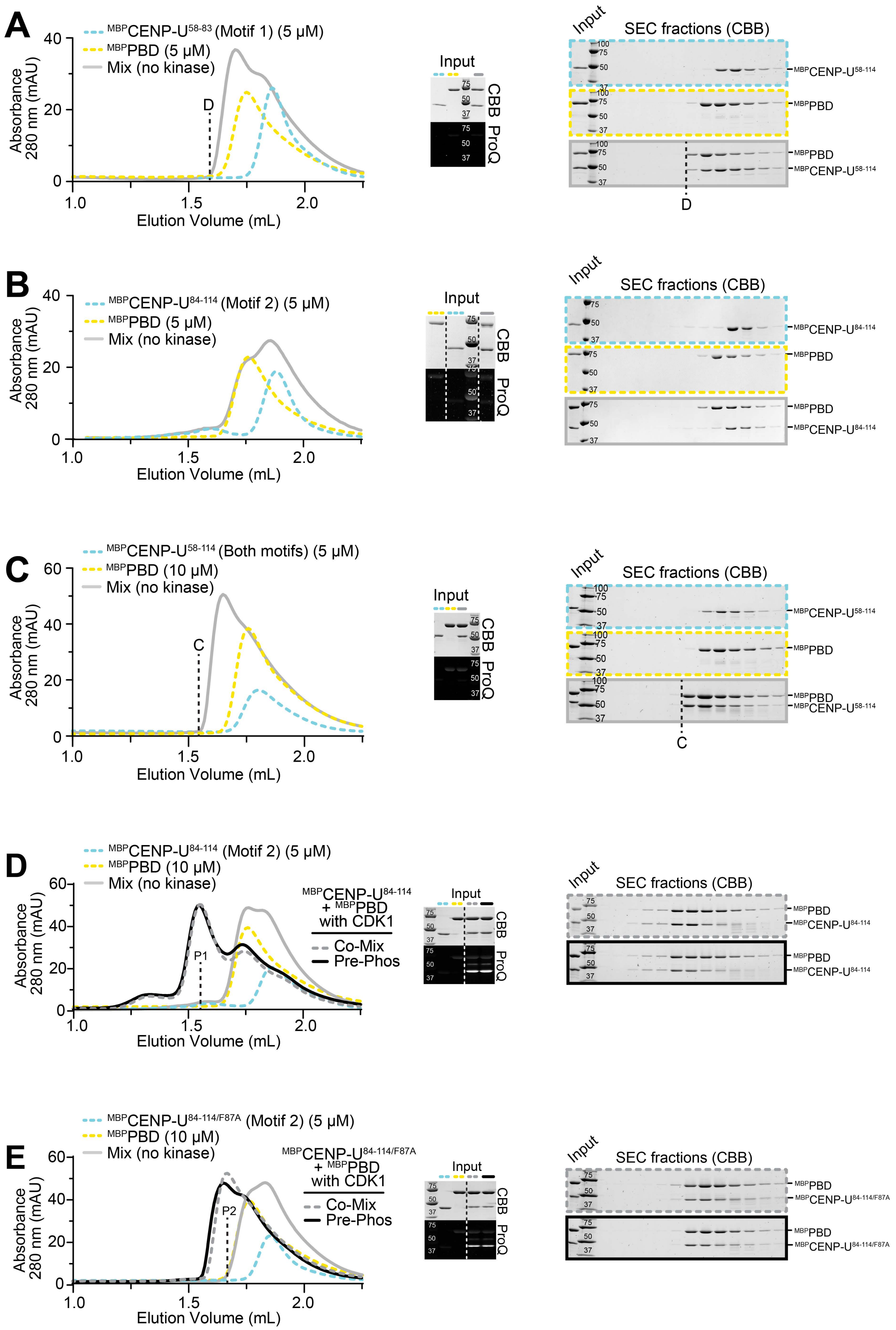
A
MBPCENP-U58-83 (Motif 1) (5 µM)
MBPPBD (5 µM)
Mix (no kinase)
Absorbance 280 nm (mAU)
Elution Volume (mL)
Input
CBB
ProQ
SEC fractions (CBB)
MBPCENP-U58-114
MBPPBD
D
B
MBPCENP-U84-114 (Motif 2) (5 µM)
MBPPBD (5 µM)
Mix (no kinase)
MBPCENP-U84-114
C
MBPCENP-U58-114 (Both motifs) (5 µM)
MBPPBD (10 µM)
Mix (no kinase)
D
MBPCENP-U84-114 (Motif 2) (5 µM)
MBPPBD (10 µM)
Mix (no kinase)
MBPCENP-U84-114 + MBPPBD with CDK1
Co-Mix
Pre-Phos
P1
E
MBPCENP-U84-114/F87A (Motif 2) (5 µM)
MBPPBD (10 µM)
Mix (no kinase)
MBPCENP-U84-114/F87A + MBPPBD with CDK1
Co-Mix
Pre-Phos
P2
MBPCENP-U84-114/F87A

Figure S1

Figure S1 ***Additional data associated with Figure 1***

(**A**) Analytical SEC profiles coupled with corresponding SDS-PAGE and Pro-Q™ analyses for the determination (along with panel B-C) that PBD has residual affinity for unphosphorylated Motif 1, but not Motif 2. D indicates the elution front of the weak complex of PBD with Motif 1. Source data are provided as a Source Data file. (**B**) No co-elution front of PBD with Motif 2 was observed. Source data are provided as a Source Data file. (**C**) C indicates the elution front for the weak complex of PBD to the unphosphorylated ^{MBP}CENP-U$^{58\text{-}114}$ (covering both Motif 1 and 2). Source data are provided as a Source Data file. (**D**) Demonstration (along with panel E) that the hydrophobic residue (F87$^{CENP\text{-}U}$) is required for stable binding between Motif 2 and PBD. P1 indicates the elution peak of the stable complex of the PBD with the phosphorylated Motif 2. Source data are provided as a Source Data file. (**E**) P2 indicates the elution peak of the weak complex of PBD with phosphorylated Motif 2 carrying the F87 $^{CENP\text{-}U}$ mutation. Both *in-situ* phosphorylation methods (see Methods) were applied for protein phosphorylation displayed in panels D and E. Source data are provided as a Source Data file.

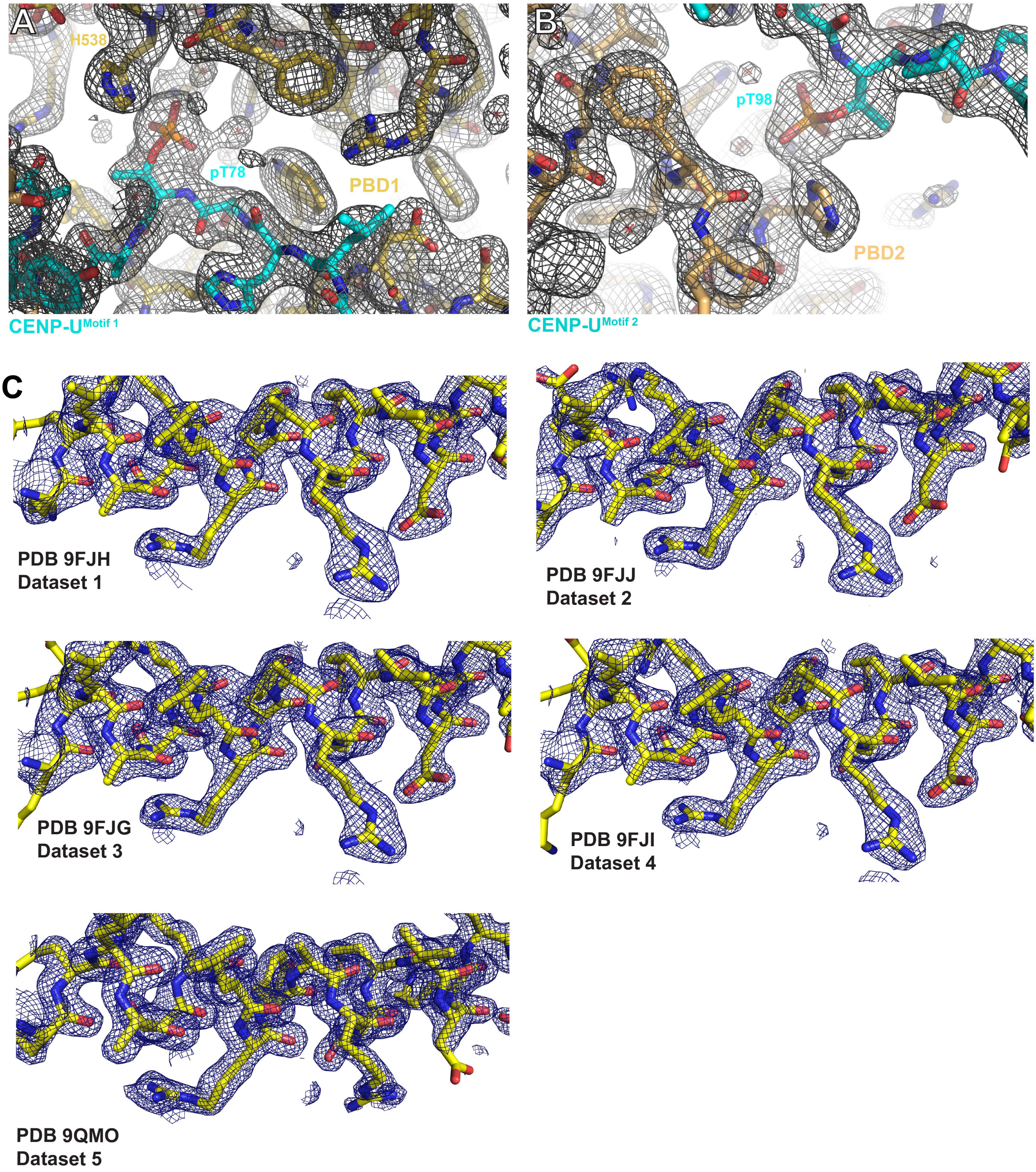
A
H538
pT78
PBD1
CENP-U^Motif 1
B
pT98
PBD2
CENP-U^Motif 2
C
PDB 9FJH
Dataset 1
PDB 9FJJ
Dataset 2
PDB 9FJG
Dataset 3
PDB 9FJI
Dataset 4
PDB 9QMO
Dataset 5

Figure S2

Figure S2 ***Additional data associated with Figure 2***

(**A**) Weighted $2F_o$-F_c electron density map calculated from dataset 1 of CENP-U Motif 1 bound to PBD1, centred at pT78. (**B**) Weighted $2F_o$-F_c electron density map calculated from dataset 1 of CENP-U Motif 1 bound to PBD1, centred at pT98. (**C**) Weighted $2F_o$-F_c electron density map of the same segment for the indicated datasets and PDB coordinates. Maps were contoured at 1.2 sigma.

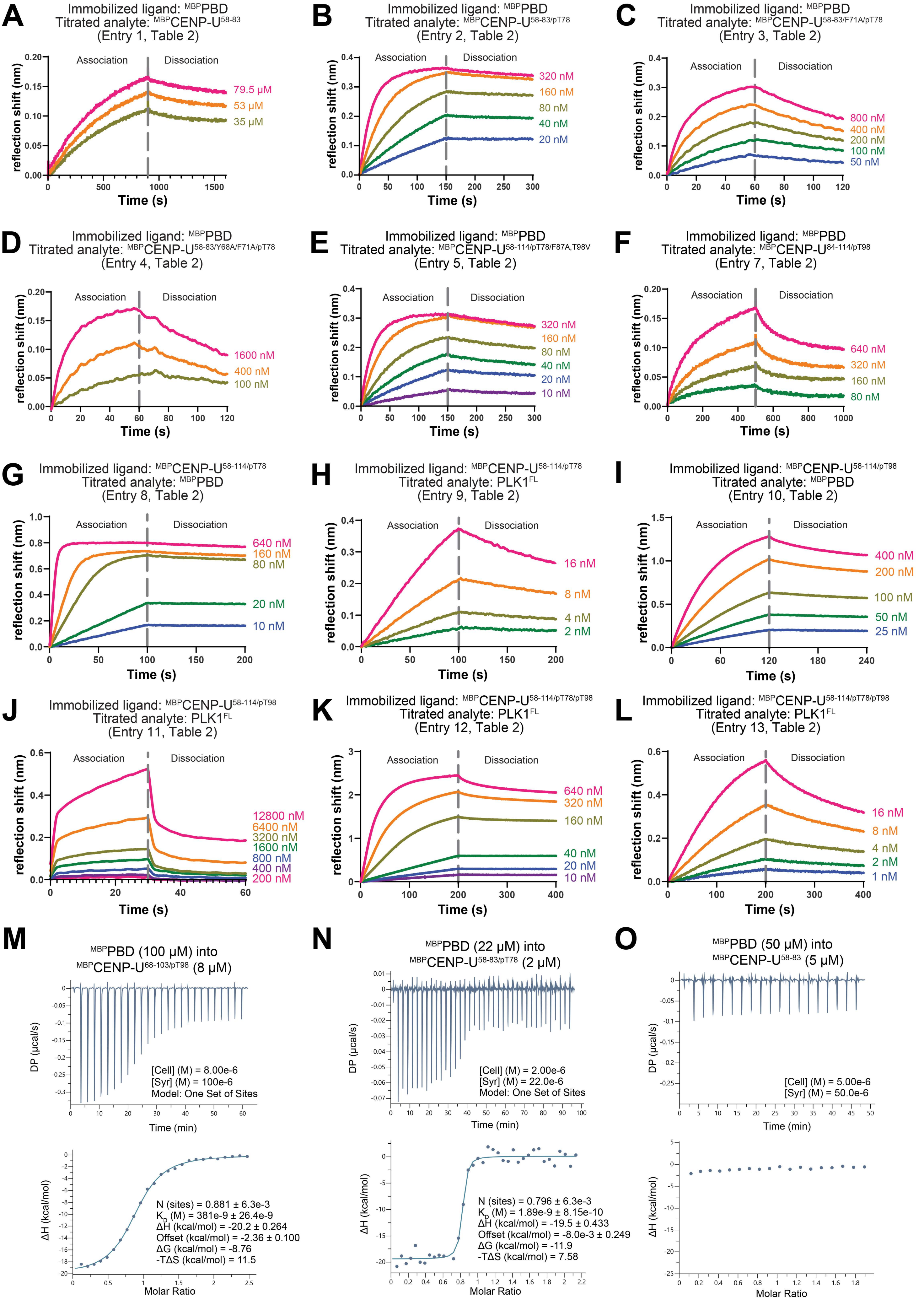
A Immobilized ligand: MBPPBD Titrated analyte: MBPCENP-U58-83 (Entry 1, Table 2)
B Immobilized ligand: MBPPBD Titrated analyte: MBPCENP-U58-83/pT78 (Entry 2, Table 2)
C Immobilized ligand: MBPPBD Titrated analyte: MBPCENP-U58-83/F71A/pT78 (Entry 3, Table 2)
D Immobilized ligand: MBPPBD Titrated analyte: MBPCENP-U58-83/Y68A/F71A/pT78 (Entry 4, Table 2)
E Immobilized ligand: MBPPBD Titrated analyte: MBPCENP-U58-114/pT78/F87A,T98V (Entry 5, Table 2)
F Immobilized ligand: MBPPBD Titrated analyte: MBPCENP-U84-114/pT98 (Entry 7, Table 2)
G Immobilized ligand: MBPCENP-U58-114/pT78 Titrated analyte: MBPPBD (Entry 8, Table 2)
H Immobilized ligand: MBPCENP-U58-114/pT78 Titrated analyte: PLK1FL (Entry 9, Table 2)
I Immobilized ligand: MBPCENP-U58-114/pT98 Titrated analyte: MBPPBD (Entry 10, Table 2)
J Immobilized ligand: MBPCENP-U58-114/pT98 Titrated analyte: PLK1FL (Entry 11, Table 2)
K Immobilized ligand: MBPCENP-U58-114/pT78/pT98 Titrated analyte: PLK1FL (Entry 12, Table 2)
L Immobilized ligand: MBPCENP-U58-114/pT78/pT98 Titrated analyte: PLK1FL (Entry 13, Table 2)
Association
Dissociation
reflection shift (nm)
Time (s)
79.5 µM
53 µM
35 µM
320 nM
160 nM
80 nM
40 nM
20 nM
800 nM
400 nM
200 nM
100 nM
50 nM
1600 nM
400 nM
100 nM
10 nM
640 nM
12800 nM
6400 nM
3200 nM
800 nM
16 nM
8 nM
4 nM
2 nM
1 nM
25 nM
M MBPPBD (100 µM) into MBPCENP-U68-103/pT98 (8 µM)
[Cell] (M) = 8.00e-6
[Syr] (M) = 100e-6
Model: One Set of Sites
N (sites) = 0.881 ± 6.3e-3
KD (M) = 381e-9 ± 26.4e-9
ΔH (kcal/mol) = -20.2 ± 0.264
Offset (kcal/mol) = -2.36 ± 0.100
ΔG (kcal/mol) = -8.76
-TΔS (kcal/mol) = 11.5
N MBPPBD (22 µM) into MBPCENP-U58-83/pT78 (2 µM)
[Cell] (M) = 2.00e-6
[Syr] (M) = 22.0e-6
Model: One Set of Sites
N (sites) = 0.796 ± 6.3e-3
KD (M) = 1.89e-9 ± 8.15e-10
ΔH (kcal/mol) = -19.5 ± 0.433
Offset (kcal/mol) = -8.0e-3 ± 0.249
ΔG (kcal/mol) = -11.9
-TΔS (kcal/mol) = 7.58
O MBPPBD (50 µM) into MBPCENP-U58-83 (5 µM)
[Cell] (M) = 5.00e-6
[Syr] (M) = 50.0e-6
DP (µcal/s)
Time (min)
ΔH (kcal/mol)
Molar Ratio

Figure S3

Figure S3 ***Additional data associated with Table 2***

(**A**-**L**) Sensorgrams of the indicated BLI experiments. Source data are provided as a Source Data file. (**M**-**O**) Thermograms of ITC measurements. Source data are provided as a Source Data file. ITC titrations of (**M**) ^{MBP}CENP-U$^{68\text{-}103/pT98}$, (**N**) ^{MBP}CENP-U$^{58\text{-}83/pT78}$, and (**O**) and ^{MBP}CENP-U$^{58\text{-}83}$ with ^{MBP}PBD. With the applied protein concentrations and buffer conditions in these ITC assays, no notable heat release was observed in the titration of the T78-site unphosphorylated ^{MBP}CENP-U$^{58\text{-}83}$ with ^{MBP}PBD, supporting single-site fitting model of the data calculation in panel M.

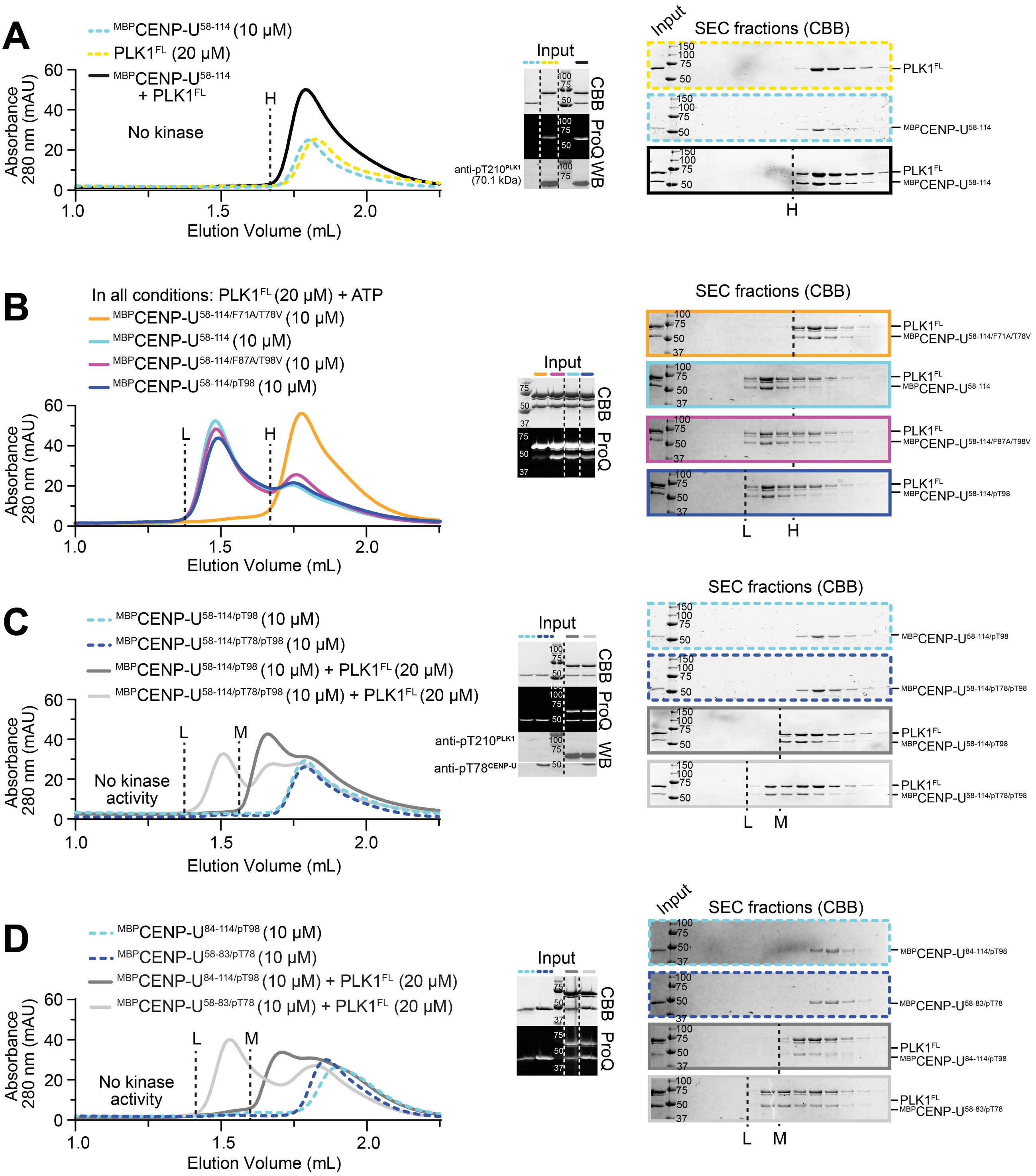
A
MBPCENP-U58-114 (10 µM)
PLK1FL (20 µM)
MBPCENP-U58-114 + PLK1FL
No kinase
H
Absorbance 280 nm (mAU)
Elution Volume (mL)
Input
CBB
ProQ
WB
anti-pT210PLK1 (70.1 kDa)
SEC fractions (CBB)
PLK1FL
MBPCENP-U58-114
B
In all conditions: PLK1FL (20 µM) + ATP
MBPCENP-U58-114/F71A/T78V (10 µM)
MBPCENP-U58-114 (10 µM)
MBPCENP-U58-114/F87A/T98V (10 µM)
MBPCENP-U58-114/pT98 (10 µM)
L
H
C
MBPCENP-U58-114/pT98 (10 µM)
MBPCENP-U58-114/pT78/pT98 (10 µM)
MBPCENP-U58-114/pT98 (10 µM) + PLK1FL (20 µM)
MBPCENP-U58-114/pT78/pT98 (10 µM) + PLK1FL (20 µM)
No kinase activity
M
anti-pT210PLK1
anti-pT78CENP-U
D
MBPCENP-U84-114/pT98 (10 µM)
MBPCENP-U58-83/pT78 (10 µM)
MBPCENP-U84-114/pT98 (10 µM) + PLK1FL (20 µM)
MBPCENP-U58-83/pT78 (10 µM) + PLK1FL (20 µM)

Figure S4

Figure S4 ***Additional data associated with Table 2***

(**A**) Analytical SEC profiles and corresponding SDS-PAGE and Pro-Q™ analyses demonstrating interactions of CENP-U pT78-Motif 1 and pT98-Motif 2 with PLK1^{FL}. Elution front H suggests no obvious affinity of PLK1^{FL} to ^{MBP}CENP-U$^{58\text{-}114}$ (covering both Motif 1 and 2). The PLK1^{FL} used in all panels A-D are active, as shown by phosphorylation of Thr210 demonstrated by Western blotting. Source data are provided as a Source Data file. (**B**) PLK1^{FL}, at 20 μM concentration, directly phosphorylates T78 of CENP-U (10 μM) upon addition of ATP, as shown by Pro-Q™ analyses. The protein concentrations are consistent in all panels. As indicated by elution front L, the CENP-U peptide and CENP-U mutated at Motif 2 (^{MBP}CENP-U$^{58\text{-}114/\,F87A/T98V}$) were phosphorylated and formed heterodimer with PLK1^{FL}. CENP-U mutated at motif 1 (^{MBP}CENP-U$^{58\text{-}114/F71A/T78V}$) can barely be phosphorylated (Pro-Q™) and showed no interaction with PLK1^{FL} (elution front H). Notably, T98-prephosphorylated CENP-U (^{MBP}CENP-U$^{58\text{-}114/pT98}$), subject to the same PLK1 phosphorylation treatment to phosphorylated T78, bound only one PLK1^{FL} (elution front L), confirming that dimerization of PLK1^{FL} on CENP-U is not as stable as dimerization of the PBD. Source data are provided as a Source Data file. (**C**) In absence of ATP-Mg^{2+}, doubly-phosphorylated CENP-U (^{MBP}CENP-U$^{58\text{-}114/pT78/pT98}$, validated by Western blotting) also bound a single PLK1^{FL} (elution front L). Notably, T98-prephosphorylated CENP-U, without phosphorylation at T78, revealed a weaker interaction with PLK1^{FL} (elution front M). Source data are provided as a Source Data file. (**D**) In contrast with the T98-prephosphorylated CENP-U (elution front M, also shown in panel C), T78-prephosphorylated CENP-U showed strong binding with PLK1^{FL} (elution front L). Source data are provided as a Source Data file.

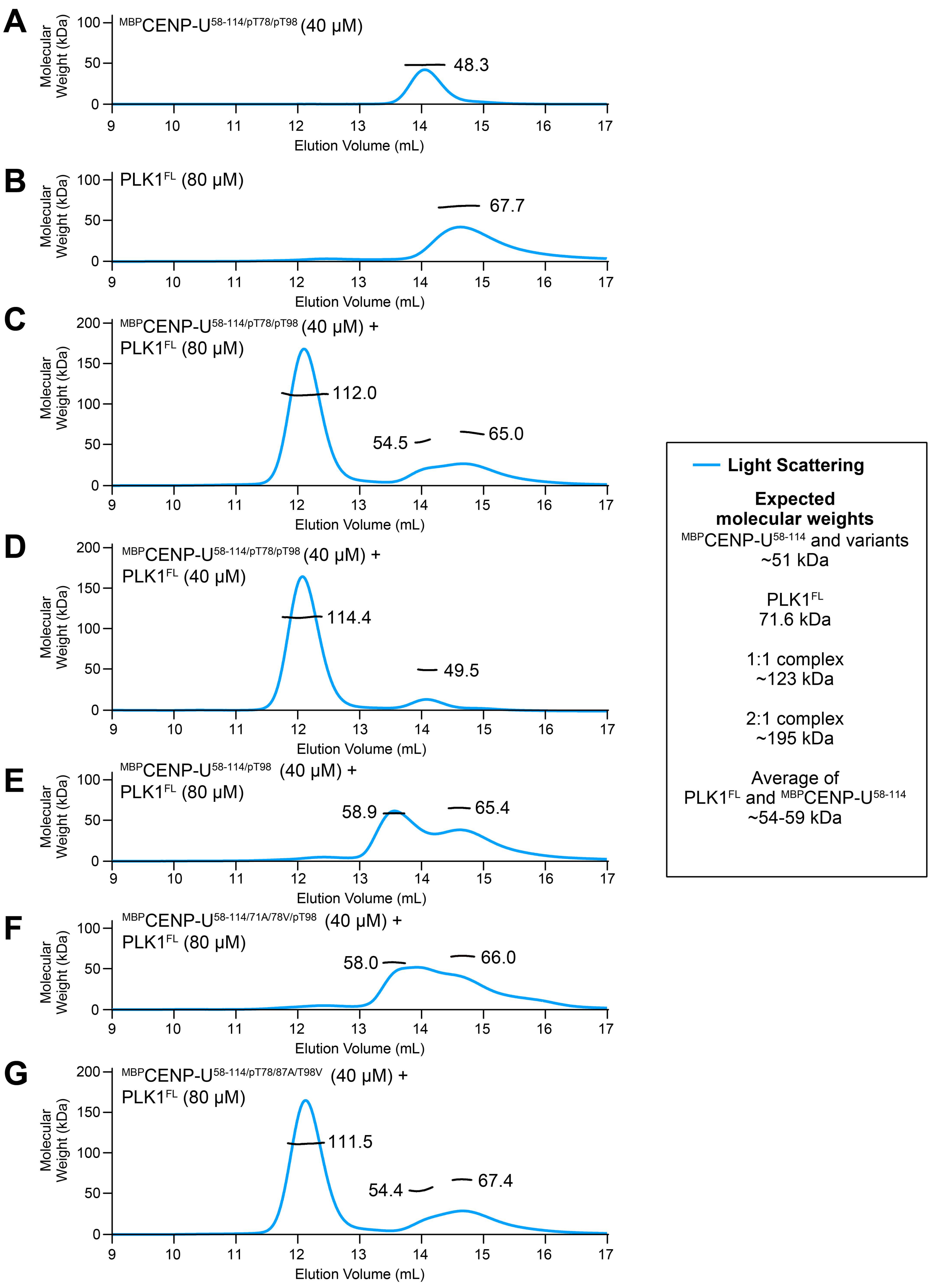
A
MBPCENP-U58-114/pT78/pT98 (40 µM)
48.3
B
PLK1FL (80 µM)
67.7
C
MBPCENP-U58-114/pT78/pT98 (40 µM) +
PLK1FL (80 µM)
112.0
54.5
65.0
D
MBPCENP-U58-114/pT78/pT98 (40 µM) +
PLK1FL (40 µM)
114.4
49.5
E
MBPCENP-U58-114/pT98 (40 µM) +
PLK1FL (80 µM)
58.9
65.4
F
MBPCENP-U58-114/71A/78V/pT98 (40 µM) +
PLK1FL (80 µM)
58.0
66.0
G
MBPCENP-U58-114/pT78/87A/T98V (40 µM) +
PLK1FL (80 µM)
111.5
54.4
67.4
Molecular Weight (kDa)
Elution Volume (mL)
Light Scattering
Expected molecular weights
MBPCENP-U58-114 and variants
~51 kDa
PLK1FL
71.6 kDa
1:1 complex
~123 kDa
2:1 complex
~195 kDa
Average of
PLK1FL and MBPCENP-U58-114
~54-59 kDa

Figure S5

Figure S5 ***Additional data associated with Table 2***

(**A**) SEC-profiles and average size-based molecular weight (MW) in SEC-MALS measurement of the pre-prepared (^{MBP}CENP-U$^{58\text{-}114/pT78/pT98}$, see also Figure S4C). The calculated MW for the monomer is 51.2 kDa (see legend). Source data are provided as a Source Data file. (**B**) SEC-MALS analysis of the PLK1^{FL} monomer. Source data are provided as a Source Data file. (**C**) SEC-MALS analysis of the doubly-phosphorylated CENP-U (panel A) with PLK1^{FL} (panel B) detected a 1:1 complex, despite a 1:2 molar mixing ratio. Source data are provided as a Source Data file. (**D**) SEC-MALS of the same sample generated at a mixing ratio of 1:1 gave a similar result. (**E**) SEC-MALS of the T98-prephosphorylated CENP-U (^{MBP}CENP-U$^{58\text{-}114/pT98}$) and PLK1^{FL} delivered a MW suggesting a weak interaction, consistent with results in Figure S4C-D. (**F**) A result similar to that obtained in panel E was obtained when Motif 1 was mutated and T98-prephosphorylated with CDK1 (^{MBP}CENP-U$^{58\text{-}114/F71A/T78V/pT98}$). Source data are provided as a Source Data file. (**G**) In contrast to the results in panel F, SEC-MALS revealed strong binding of PLK1^{FL} mixed 1:1 with CENP-U prephosphorylated on T78 and mutated on Motif 2 (^{MBP}CENP- U$^{58\text{-}114/pT78/F87A/T98V}$). Source data are provided as a Source Data file.

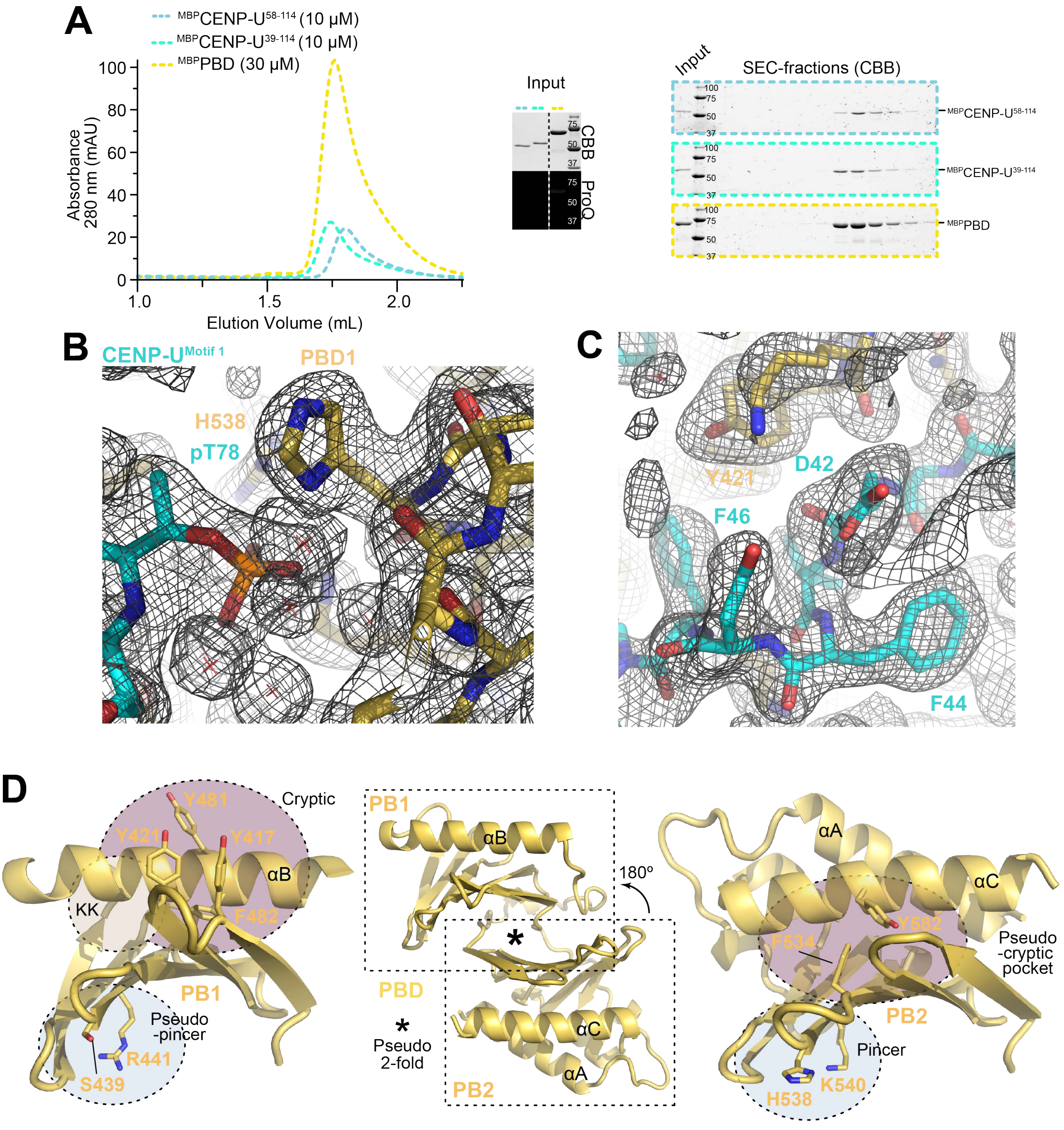
A
MBPCENP-U58-114 (10 μM)
MBPCENP-U39-114 (10 μM)
MBPPBD (30 μM)
Absorbance 280 nm (mAU)
Elution Volume (mL)
Input
CBB
ProQ
SEC-fractions (CBB)
MBPCENP-U58-114
MBPCENP-U39-114
MBPPBD
B
CENP-UMotif 1
PBD1
H538
pT78
C
D42
Y421
F46
F44
D
Cryptic
Y481
Y421
Y417
αB
KK
F482
PB1
Pseudo -pincer
R441
S439
PB1
αB
180º
PBD
Pseudo 2-fold
PB2
αC
αA
αA
αC
Y582
F534
Pseudo -cryptic pocket
PB2
Pincer
H538
K540

Figure S6

Figure S6 ***Additional data associated with Figure 3***

(**A**) Analytical SEC profiles coupled with corresponding SDS-PAGE and Pro-Q™ analyses of control protein samples used for the experiments in Figure 3B. Source data are provided as a Source Data file. (**B**) Weighted $2F_o$-F_c electron density maps of interacting regions in the crystallized complex of N-terminal extended ^{MBP}CENP-U$^{39\text{-}114}$ in compex with two PBDs (Table 1, dataset 2; Figure 3C). T78$^{CENP\text{-}U}$ phosphorylation was clearly detected. The phosphorylated side chain interacts with the canonical pincer residues of the PBD. (**C**) The weighted $2F_o$-F_c electron density map of the N-terminal extended region (residues 39-47) of CENP-U demonstrates extensive contacts with the PBD, including sandwiching at a crystal contact (not shown). (**D**) PB1 and PB2 are related by pseudo-2-fold symmetry. We superposed them as explained in the middle panel and show them here in the same orientation on the left (PB1) and right (PB2). This identifies aromatic residues (F534 and Y582) positioned similarly to those in the cryptic pocket, defining the pseudo-cryptic pocket. It also identifies residues equivalent to the pincer, S439 and R441. Helices are marked to help orientation.

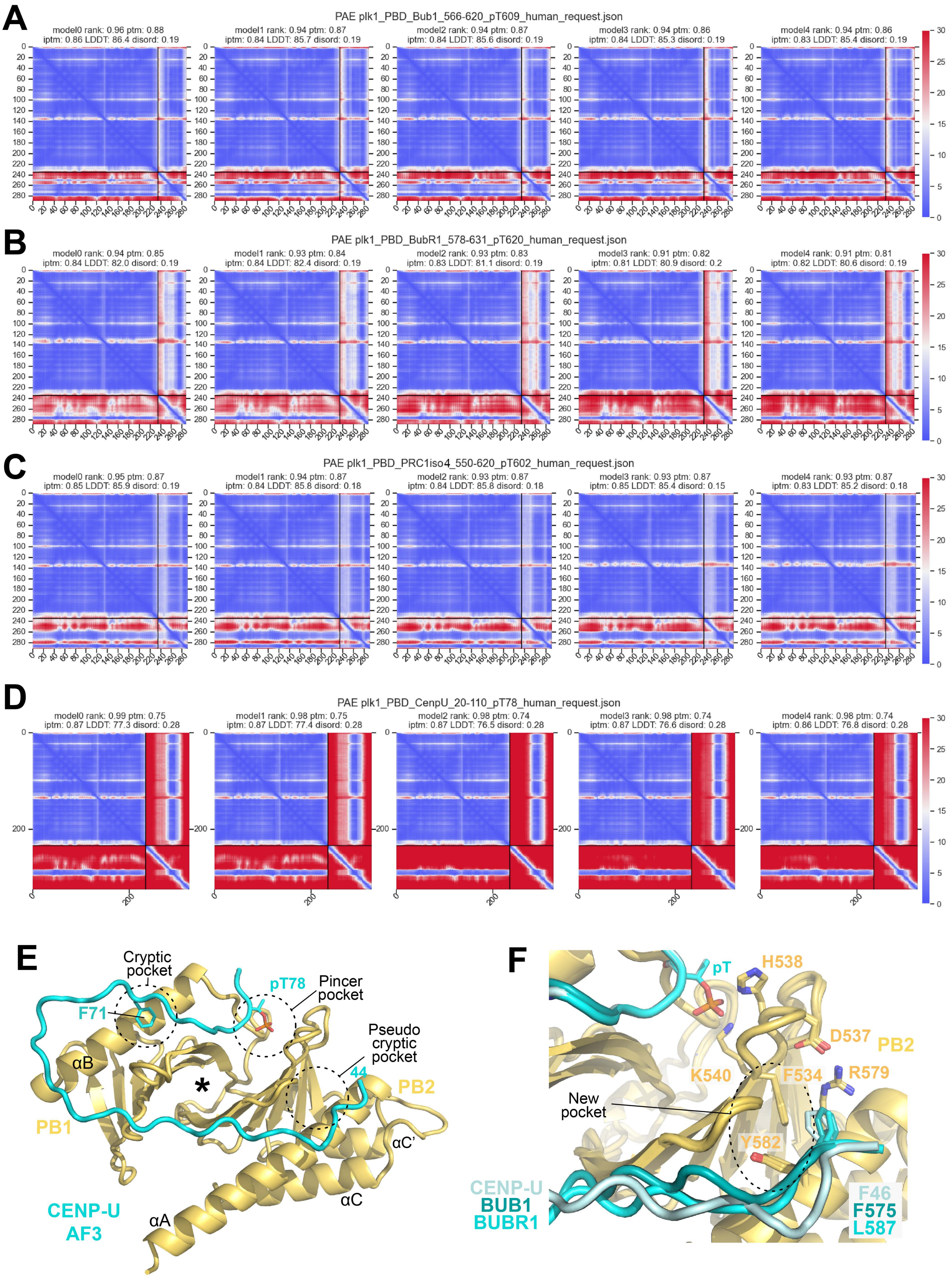
A
PAE plk1_PBD_Bub1_566-620_pT609_human_request.json
model0 rank: 0.96 ptm: 0.88
iptm: 0.86 LDDT: 86.4 disord: 0.19
model1 rank: 0.94 ptm: 0.87
iptm: 0.84 LDDT: 85.7 disord: 0.19
model2 rank: 0.94 ptm: 0.87
iptm: 0.84 LDDT: 85.6 disord: 0.19
model3 rank: 0.94 ptm: 0.86
iptm: 0.84 LDDT: 85.3 disord: 0.19
model4 rank: 0.94 ptm: 0.86
iptm: 0.83 LDDT: 85.4 disord: 0.19
B
PAE plk1_PBD_BubR1_578-631_pT620_human_request.json
model0 rank: 0.94 ptm: 0.85
iptm: 0.84 LDDT: 82.0 disord: 0.19
model1 rank: 0.93 ptm: 0.84
iptm: 0.84 LDDT: 82.4 disord: 0.19
model2 rank: 0.93 ptm: 0.83
iptm: 0.83 LDDT: 81.1 disord: 0.19
model3 rank: 0.91 ptm: 0.82
iptm: 0.81 LDDT: 80.9 disord: 0.2
model4 rank: 0.91 ptm: 0.81
iptm: 0.82 LDDT: 80.6 disord: 0.19
C
PAE plk1_PBD_PRC1iso4_550-620_pT602_human_request.json
model0 rank: 0.95 ptm: 0.87
iptm: 0.85 LDDT: 85.9 disord: 0.19
model1 rank: 0.94 ptm: 0.87
iptm: 0.84 LDDT: 85.8 disord: 0.18
model2 rank: 0.93 ptm: 0.87
iptm: 0.84 LDDT: 85.8 disord: 0.18
model3 rank: 0.93 ptm: 0.87
iptm: 0.85 LDDT: 85.4 disord: 0.15
model4 rank: 0.93 ptm: 0.87
iptm: 0.83 LDDT: 85.2 disord: 0.18
D
PAE plk1_PBD_CenpU_20-110_pT78_human_request.json
model0 rank: 0.99 ptm: 0.75
iptm: 0.87 LDDT: 77.3 disord: 0.28
model1 rank: 0.98 ptm: 0.75
iptm: 0.87 LDDT: 77.4 disord: 0.28
model2 rank: 0.98 ptm: 0.74
iptm: 0.87 LDDT: 76.5 disord: 0.28
model3 rank: 0.98 ptm: 0.74
iptm: 0.87 LDDT: 76.6 disord: 0.28
model4 rank: 0.98 ptm: 0.74
iptm: 0.86 LDDT: 76.8 disord: 0.28
E
Cryptic pocket
pT78
Pincer pocket
F71
Pseudo cryptic pocket
44
αB
PB2
PB1
αC'
αC
CENP-U
AF3
αA
F
pT
H538
D537
PB2
K540
F534
R579
New pocket
Y582
CENP-U
BUB1
BUBR1
F46
F575
L587

Figure S7

Figure S7 ***Additional data associated with Figure 4***

(**A**-**D**) AF PAE scores for the indicated predictions displayed in Figure 5. (**E-F**) AF prediction of CENP-U bound to the PBD and comparison with the BUB1, BUBR1, and PRC1 predictions. AF positions the N-terminal region of CENP-U differently from what we observe in the crystal structure discussed in the context of Figure 3. The chain is far more extended and F46 of CENP-U is predicted to occupy the pseudo-cryptic pocket similar to the equivalent residues of BUBR1 or BUBR1. A completely conserved salt bridge involving D537 and R579 of the PBD in the proximity of the pseudo-cryptic pocket is shown. Although AF might reach its limits here, the prediction could suggest that CENP-U may use additional N-terminal residues to extend along the PBD.

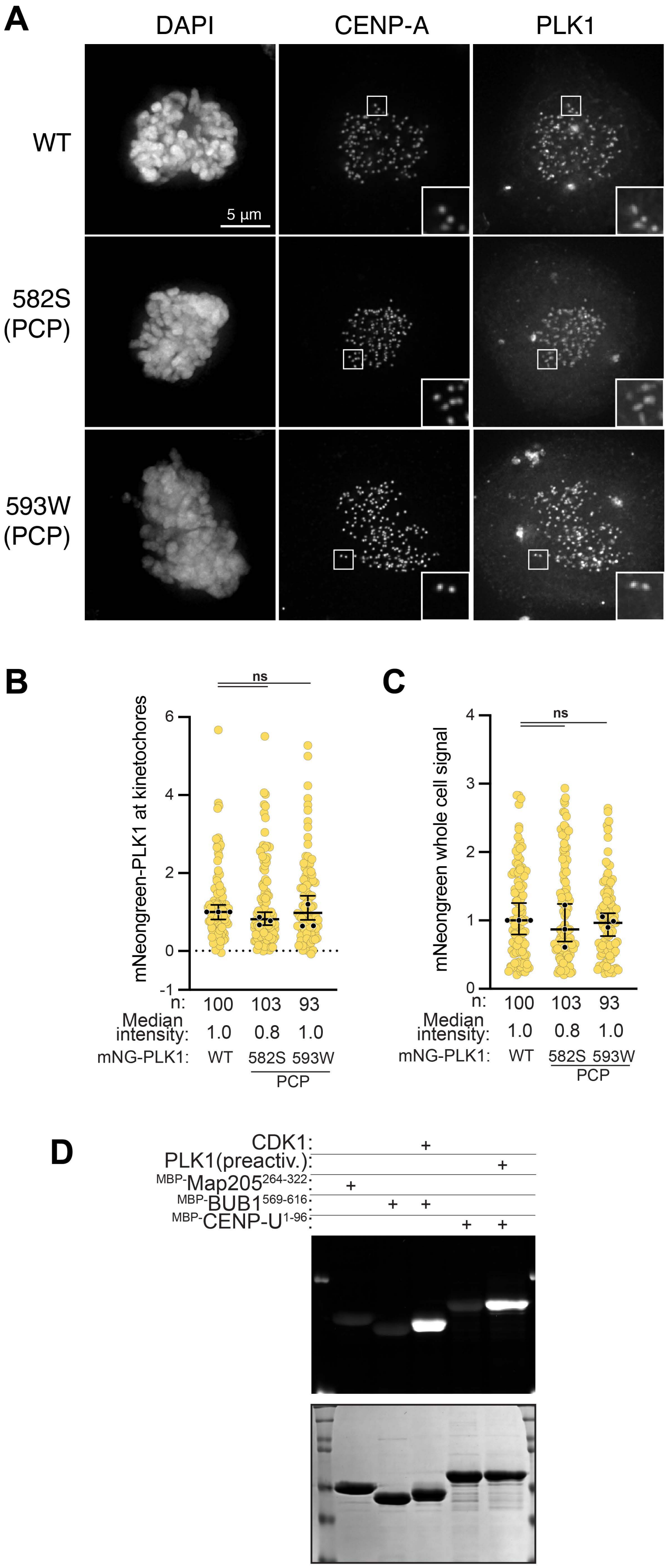
A
DAPI
CENP-A
PLK1
WT
5 µm
582S
(PCP)
593W
(PCP)
B
ns
mNeongreen-PLK1 at kinetochores
n: 100 103 93
Median intensity: 1.0 0.8 1.0
mNG-PLK1: WT 582S 593W
PCP
C
ns
mNeongreen whole cell signal
n: 100 103 93
Median intensity: 1.0 0.8 1.0
mNG-PLK1: WT 582S 593W
PCP
D
CDK1:
PLK1(preactiv.):
MBP-Map205264-322:
MBP-BUB1569-616:
MBP-CENP-U1-96:

Figure S8

Figure S8 ***Additional data associated with Figure 4***

(**A**) Subcellular localization of an mNeonGreen-fused PLK1 transgene and the indicated mutants in mitotic HeLa cells. The first row with the wild type transgene is also shown in Figure 4E as samples are part of the same experiment. (**B**-**C**) Quantification of the experiments in panel A. The black bars represent the median of three replicates, and the vertical bar 95 % confidence interval. Yellow dots represent either kinetochore or whole cell mNeongreen signal relative to the control condition (median set to 1). Black dots are medians of the single replicate. Statistical analysis was performed using the Kruskal-Wallis test as described in Methods. To convert P values into an asterisk-based significance system, we used the default GraphPad Prism convention: not significant (ns) P > 0.05; *P ≤ 0.05 but >0.01; **P ≤ 0.01 but > 0.001; ***P ≤ 0.001 but >0.0001; and ****P ≤ 0.0001. The exact P values for pairs of conditions in (B) were: WT vs. Y582S > 0.05; WT vs. S593W > 0.05. The exact P values for pairs of conditions in (C) were: WT vs. Y582S > 0.05; WT vs. S593W > 0.05. Source data are provided as a Source Data file. (**D**) In the upper panel, Pro-Q^{TM} Diamond Phosphoprotein staining of indicated bands after CDK1 or PLK1 phosphorylation. The corresponding Coomassie-strained gel is shown below.

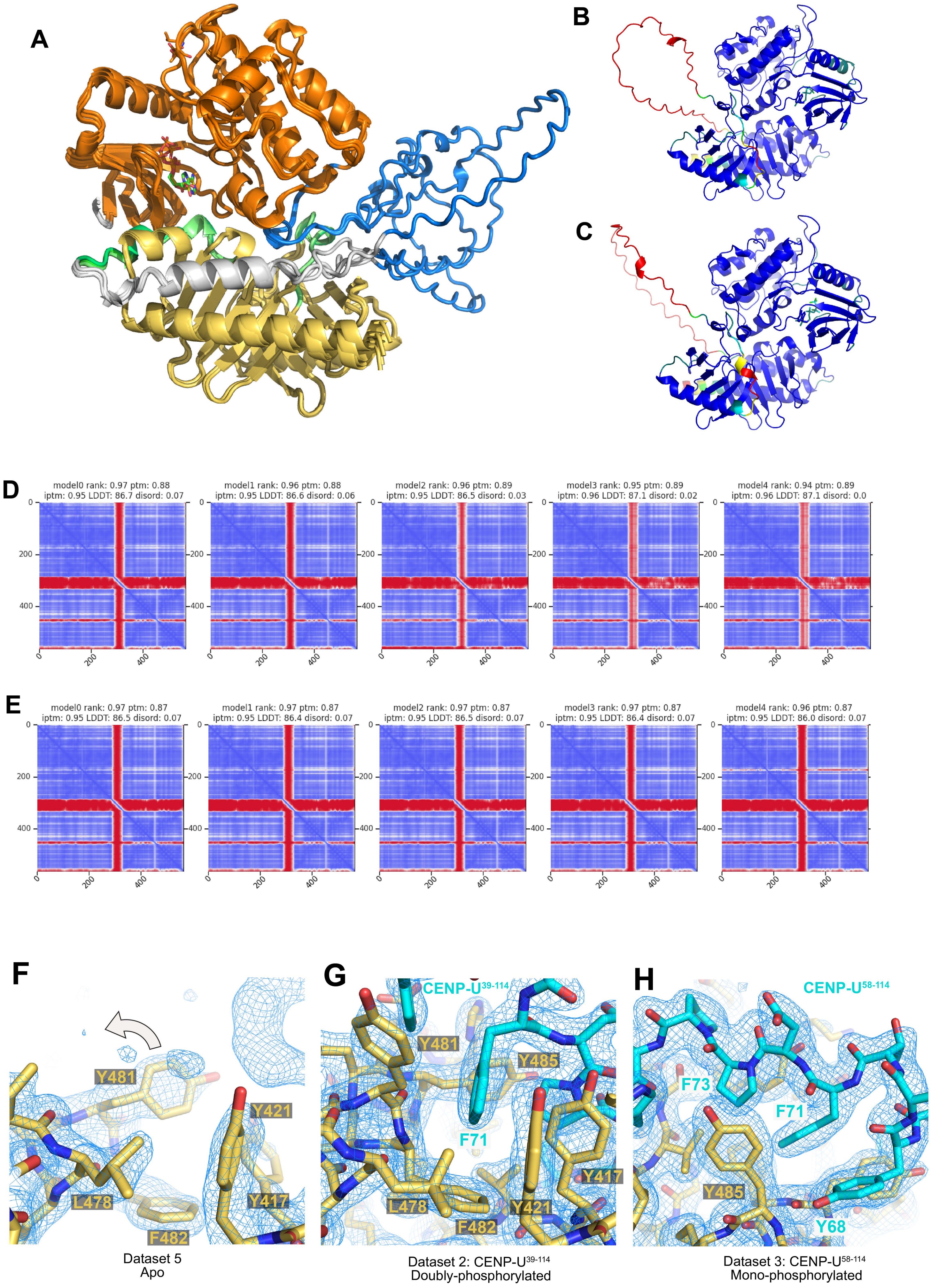
A
B
C
D
model0 rank: 0.97 ptm: 0.88
iptm: 0.95 LDDT: 86.7 disord: 0.07
model1 rank: 0.96 ptm: 0.88
iptm: 0.95 LDDT: 86.6 disord: 0.06
model2 rank: 0.96 ptm: 0.89
iptm: 0.95 LDDT: 86.5 disord: 0.03
model3 rank: 0.95 ptm: 0.89
iptm: 0.96 LDDT: 87.1 disord: 0.02
model4 rank: 0.94 ptm: 0.89
iptm: 0.96 LDDT: 87.1 disord: 0.0
E
model0 rank: 0.97 ptm: 0.87
iptm: 0.95 LDDT: 86.5 disord: 0.07
model1 rank: 0.97 ptm: 0.87
iptm: 0.95 LDDT: 86.4 disord: 0.07
model2 rank: 0.97 ptm: 0.87
iptm: 0.95 LDDT: 86.5 disord: 0.07
model3 rank: 0.97 ptm: 0.87
iptm: 0.95 LDDT: 86.4 disord: 0.07
model4 rank: 0.96 ptm: 0.87
iptm: 0.95 LDDT: 86.0 disord: 0.07
F
Y481
Y421
L478
Y417
F482
Dataset 5
Apo
G
CENP-U39-114
Y481
Y485
F71
Y417
L478
Y421
F482
Dataset 2: CENP-U39-114
Doubly-phosphorylated
H
CENP-U58-114
F73
F71
Y485
Y68
Dataset 3: CENP-U58-114
Mono-phosphorylated

Figure S9

Figure S9 ***Additional data associated with Figures 6 and 7***

(**A**) Superposition of best AF3 models for four predictions of full-length HsPLK1, including predictions for Thr210 phosphorylated and unphosphorylated PLK1 (two predictions), and the same samples with or without ATP modelled in the active site (two additional predictions). The superposition demonstrates that all four predictions converge on very similar structures except for the disordered IDL loop. (**B**-**C**) LDDT scores mapped onto cartoon models of two of the predictions (with ATP in panel B and with ATP and with pThr210 in panel C). (**D**-**E**) PAE scores for the best five models, in the same order as in panels B-C. LDDT scores and model qualities for the two additional predictions (without ATP) were essentially identical. (**F**) Position of the side chain of Tyr481 at the mouth of the cryptic pocket in the structure of the PBD calculated from Dataset 5 in absence of bound peptide. The arrow indicates the movement of the side chain of Tyr481 required to open the pocket and allow its occupation. (**G**) The side chain of Phe71 in Motif 1 in the cryptic pocket. The new position of Tyr481 is indicated. There is excellent electron density documenting the structural change. (**H**) A view of the cryptic pocket after an approximately 180° rotation from the view in panel G.

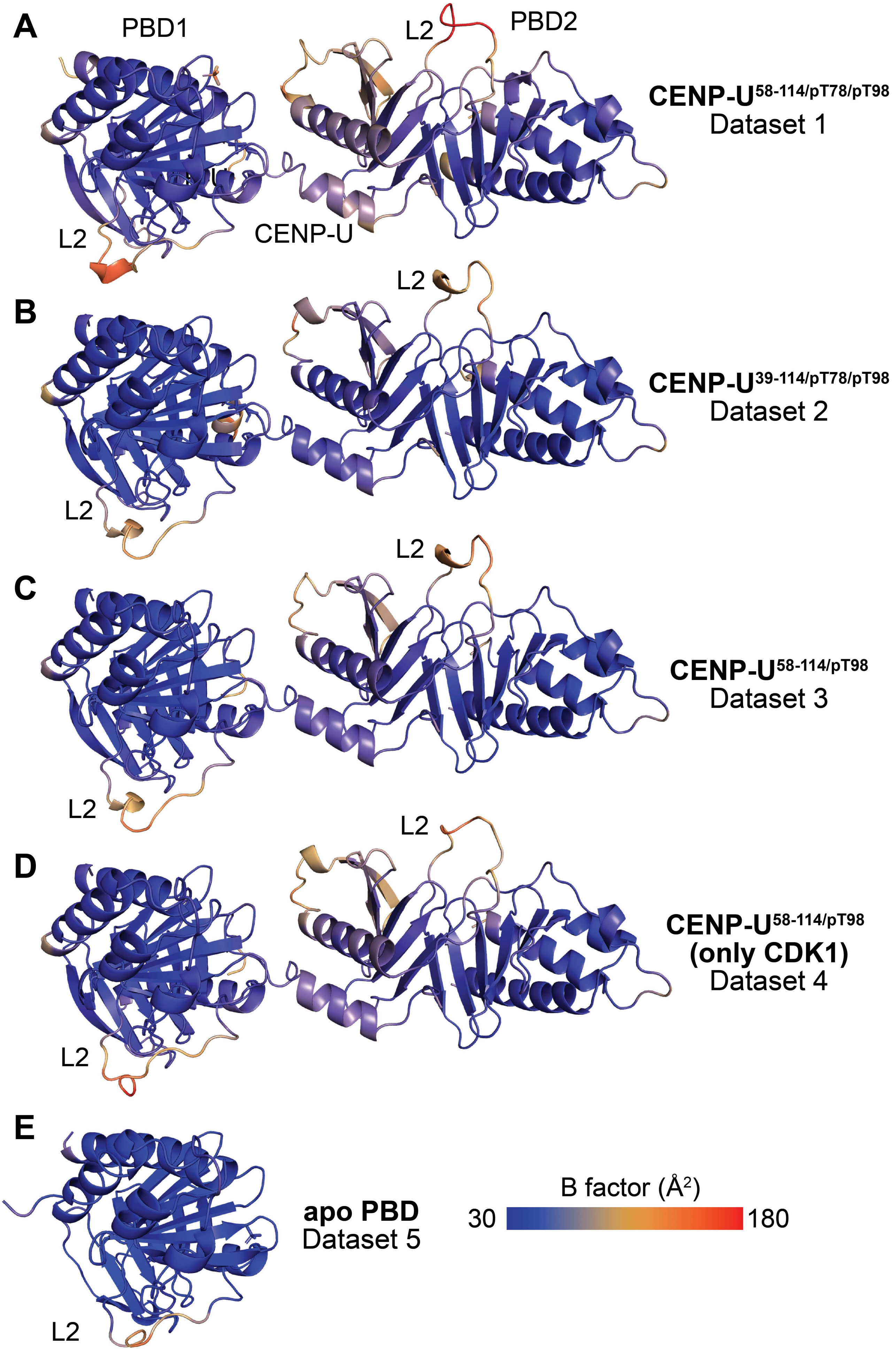
A
PBD1
L2
PBD2
CENP-U58-114/pT78/pT98
Dataset 1
L2
CENP-U
B
L2
CENP-U39-114/pT78/pT98
Dataset 2
L2
C
L2
CENP-U58-114/pT98
Dataset 3
L2
D
L2
CENP-U58-114/pT98
(only CDK1)
Dataset 4
L2
E
apo PBD
Dataset 5
B factor (Å2)
30
180
L2

Figure S10

Figure S10 ***Additional data associated with Figure 6***

(**A**-**E**) Distribution of atomic B-factors on the indicated experimental models. L2 loops in PBD1 and PBD2 are among the most mobile regions of the structures.

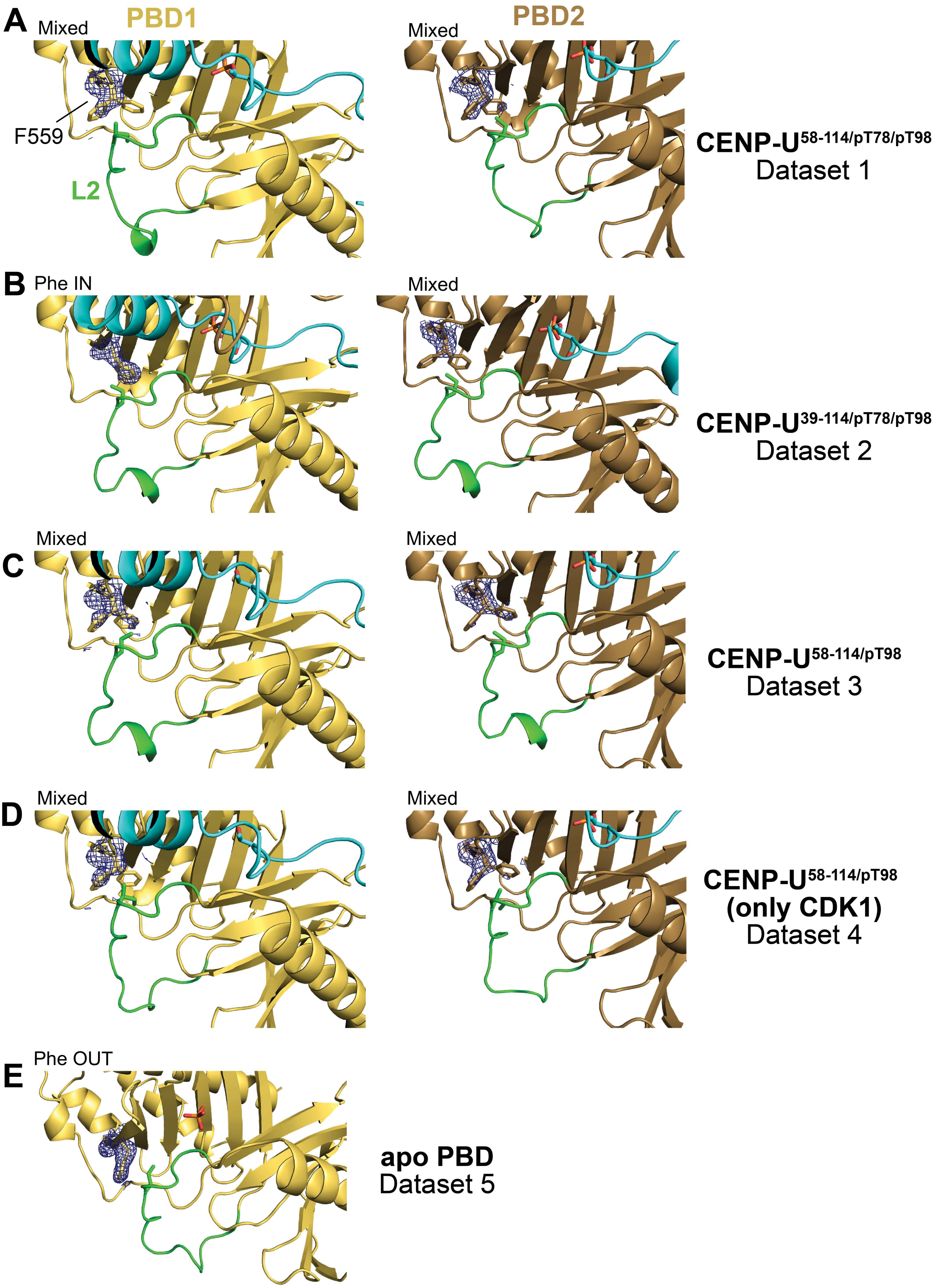
A
Mixed
PBD1
F559
L2
PBD2
Mixed
CENP-U58-114/pT78/pT98
Dataset 1
B
Phe IN
Mixed
CENP-U39-114/pT78/pT98
Dataset 2
C
Mixed
Mixed
CENP-U58-114/pT98
Dataset 3
D
Mixed
Mixed
CENP-U58-114/pT98
(only CDK1)
Dataset 4
E
Phe OUT
apo PBD
Dataset 5

Figure S11

Figure S11 ***Additional data associated with Figure 7***

(**A**-**E**) Electron density and modelling of the side chain of Phe599 (F599) in PBD1 and PBD2 of the indicated datasets. "Mixed" means that each of the two conformations, Phe IN and Phe OUT, is represented, to various levels, in the electron density, even if one of the forms predominates. "Pure" Phe IN or Phe OUT conformations are only observed in PBD1 in Dataset 2 and apo PBD in Dataset 5.
