# Supplementary material for: Molecular anatomy of PLK1 master docking motifs
Source: Nat Commun. 2026 May 11;17:4228. doi: 10.1038/s41467-026-73038-8 (PMC13161223; doi:10.1038/s41467-026-73038-8)
Supplement: Supplementary file 2 — Reporting Summary [file 41467_2026_73038_MOESM2_ESM.pdf]

Corresponding author(s): Andrea Musacchio
Last updated by author(s): Jan 30, 2026

Reporting Summary

Nature Portfolio wishes to improve the reproducibility of the work that we publish. This form provides structure for consistency and transparency in reporting. For further information on Nature Portfolio policies, see our Editorial Policies and the Editorial Policy Checklist.

Statistics

For all statistical analyses, confirm that the following items are present in the figure legend, table legend, main text, or Methods section.

n/a	Confirmed	
☐	☒	The exact sample size (*n*) for each experimental group/condition, given as a discrete number and unit of measurement
☐	☒	A statement on whether measurements were taken from distinct samples or whether the same sample was measured repeatedly
☐	☒	The statistical test(s) used AND whether they are one- or two-sided<br>*Only common tests should be described solely by name; describe more complex techniques in the Methods section.*
☒	☐	A description of all covariates tested
☒	☐	A description of any assumptions or corrections, such as tests of normality and adjustment for multiple comparisons
☐	☒	A full description of the statistical parameters including central tendency (e.g. means) or other basic estimates (e.g. regression coefficient) AND variation (e.g. standard deviation) or associated estimates of uncertainty (e.g. confidence intervals)
☐	☒	For null hypothesis testing, the test statistic (e.g. *F*, *t*, *r*) with confidence intervals, effect sizes, degrees of freedom and *P* value noted<br>*Give P values as exact values whenever suitable.*
☒	☐	For Bayesian analysis, information on the choice of priors and Markov chain Monte Carlo settings
☒	☐	For hierarchical and complex designs, identification of the appropriate level for tests and full reporting of outcomes
☒	☐	Estimates of effect sizes (e.g. Cohen's *d*, Pearson's *r*), indicating how they were calculated

Our web collection on statistics for biologists contains articles on many of the points above.

Software and code

Policy information about availability of computer code

Data collection

Image Lab Bio-rad https://www.bio-rad.com/de-de/product/image-lab-software?ID=KRE6P5E8Z
XDS 2023 https://wiki.uni-konstanz.de/xds
Octet BLI Discover 13.0 software (Sartorius, https://www.sartorius.com/en/products/biolayer-interferometry)
PEAQ-ITC Control Software V1.41 (Malvern Panalytical, https://www.malvernpanalytical.com/en/products/product-range/microcal-range/microcal-itc-range/microcal-peaq-itc)
LC-MS Data Acquisition Software (Thermo Fisher Scientific)
SoftWoRx (GE Healthcare)

Data analysis	Phenix 1.21.2_5419 (Adams, P.D. et al.) and Coot 0.9 (Emsley, P. et al.)<br>AlphaFold-Multimer v3.2.1 (Evans et al., 2021)<br>ChimeraX-1.5 (Pettersen et al., 2021)<br>Pymol 2.60 software23 (Schrödinger LLC, New York, NY).<br>GraphPad Prism Version 10 (GraphPad Software Inc http://www.graphpad.com)<br>Astra software for SEC-MALS (Wyatt, version 7.3)<br>Octet Analysis 13.0 (Sartorius, https://www.sartorius.com/en/products/biolayer-interferometry)<br>PEAQ-ITC Analysis Software V1.41 (Malvern Panalytical, https://www.malvernpanalytical.com/en/products/product-range/microcal-range/microcal-itc-range/microcal-peaq-itc)<br>MaxQuant version 2.2.0.0 (Cox, J. & Mann, M.)<br>Skyline (Schilling, B. et al.)<br>Fiji Version 2.0.0-rc-69/1.52p (Schindelin et al., 2012 http://imageJ.nih.gov/ij/)

For manuscripts utilizing custom algorithms or software that are central to the research but not yet described in published literature, software must be made available to editors and reviewers. We strongly encourage code deposition in a community repository (e.g. GitHub). See the Nature Portfolio guidelines for submitting code & software for further information.

Data

Policy information about availability of data

All manuscripts must include a data availability statement. This statement should provide the following information, where applicable:

- Accession codes, unique identifiers, or web links for publicly available datasets
- A description of any restrictions on data availability
- For clinical datasets or third party data, please ensure that the statement adheres to our policy

The model coordinates of protein structures solved in this study have been stored in the Protein Data Bank (PDB) and are accessible through PDB_id or PDB_doi: dataset 1 (9FJH, https://doi.org/10.2210/pdb9FJH/pdb), dataset 2 (9FJJ, https://doi.org/10.2210/pdb9FJJ/pdb), dataset 3 (9FJG, https://doi.org/10.2210/pdb9FJG/pdb), dataset 4 (9FJI, https://doi.org/10.2210/pdb9FJI/pdb) and dataset 5 (9QMO, https://doi.org/10.2210/pdb9qmo/pdb, awaiting release upon paper publication). The corresponding diffraction data were collected and stored at Swiss Light Source at the Paul Scherrer Institut (dataset 1, 3-5) or at European Synchrotron Radiation Facility (dataset 2). Other data are stored in the lab and available from the corresponding author upon reasonable request.

Research involving human participants, their data, or biological material

Policy information about studies with human participants or human data. See also policy information about sex, gender (identity/presentation), and sexual orientation and race, ethnicity and racism.

Reporting on sex and gender	Not applicable
Reporting on race, ethnicity, or other socially relevant groupings	Not applicable
Population characteristics	Not applicable
Recruitment	Not applicable
Ethics oversight	Not applicable

Note that full information on the approval of the study protocol must also be provided in the manuscript.

Field-specific reporting

Please select the one below that is the best fit for your research. If you are not sure, read the appropriate sections before making your selection.

☒ Life sciences ☐ Behavioural & social sciences ☐ Ecological, evolutionary & environmental sciences

For a reference copy of the document with all sections, see nature.com/documents/nr-reporting-summary-flat.pdf

Life sciences study design

All studies must disclose on these points even when the disclosure is negative.

Sample size	For cell biology experiments, a sample between 21 and 52 cells was analyzed and reported in the final quantification for each condition and per biological replicate, leading to a final sample size of at least 75 cells per condition.
Data exclusions	An upper and lower threshold based on whole cell intensity signal in the 488 nm channel was used to look at homogenously expressing cells
Replication	We indicate the number of biological/technical replicates of each experiment in the relevant figure legends. For cell biology experiments, three biological replicates were performed. In Mass spectrometry analysis, five replicates were performed.

Randomization	For each immuno-fluorescence analysis, cells were selected based on whole cell fluorescence intensity in the 488 nm channel. The final readout of interest (kinetochore localization of PLK1) was more accurately measured by knowing that selected cells expressed the PLK1 transgene homogenously.
Blinding	The investigators were not blinded during data collection. The same investigators carried out the data collection and data analysis processes

Reporting for specific materials, systems and methods

We require information from authors about some types of materials, experimental systems and methods used in many studies. Here, indicate whether each material, system or method listed is relevant to your study. If you are not sure if a list item applies to your research, read the appropriate section before selecting a response.

Materials & experimental systems

n/a	Involved in the study	
☐	☒	Antibodies
☐	☒	Eukaryotic cell lines
☒	☐	Palaeontology and archaeology
☒	☐	Animals and other organisms
☒	☐	Clinical data
☒	☐	Dual use research of concern
☒	☐	Plants

Methods

n/a	Involved in the study	
☒	☐	ChIP-seq
☒	☐	Flow cytometry
☒	☐	MRI-based neuroimaging

Antibodies

Antibodies used	anti-CENP-A (mouse monoclonal, GeneTex GTX13939, 1:500)<br>anti–PBIP-1 (phospho-T78) (rabbit polyclonal, Abcam, Cambridge, 1:1000)<br>anti-PLK1 (phospho-T210) (mouse monoclonal, Biolegend #629801, 1:1000)<br><br>anti-mouse Rodamine Red (goat, Jackson Immunoresearch 115-295-003, 1:200)<br><br>anti-mouse HRP-conjugated (NXA931, Amersham, 1:5000)<br>anti-rabbit HRP-conjugated (NA934V, Amersham, 1:5000)
Validation	Primary antibodies used for IF recognized a signal that disappeared upon RNAi depletion or in immunoblot on purified recombinant proteins.<br><br>anti-CENP-A (mouse monoclonal, GeneTex, 1:500): https://www.genetex.com/Product/Detail/CENPA-antibody-3-19/GTX13939?srsltid=AfmBOooPTwY--SViTciBcol8EH6lTp0wlQs7DuNHG34FfRvuHkuwIE4i<br>anti–PBIP-1 (phosphor-T78) (rabbit polyclonal, Abcam, Cambridge, 1:1000): discontinued (https://www.abcam.com/en-us/products/primary-antibodies/mlf1-interacting-protein-pbip1-phospho-t78-antibody-ab115570)<br>anti-PLK1 (phospho-T210) (mouse monoclonal, Biolegend #629801, 1:1000): https://www.biolegend.com/fi-fi/products/purified-anti-plk-1-phospho-thr210-antibody-3052?pdf=true&displayInline=true&leftRightMargin=15&topBottomMargin=15&filename=Purified%20anti-PLK-1%20Phospho%20(Thr210)%20Antibody.pdf&v=20260103043746<br><br>anti-mouse Rodamine Red (goat, Jackson Immunoresearch, 1:200): https://www.jacksonimmuno.com/catalog/products/115-295-003<br><br>anti-mouse HRP-conjugated (NXA931, Amersham, 1:5000): https://www.cytivalifesciences.com/en/us/shop/protein-analysis/blotting-and-detection/blotting-standards-and-reagents/amersham-ecl-hrp-conjugated-antibodies-p-06260<br>anti-rabbit HRP-conjugated (NA934V, Amersham, 1:5000): https://www.cytivalifesciences.com/en/us/search#q=Amersham%20ECL%20rabbit%20IgG&t=coveo5819fbca

Eukaryotic cell lines

Policy information about cell lines and Sex and Gender in Research

Cell line source(s)	-Sf9 cells (GibcoTMInvitrogen Corporation, Cat. No. 11496-015)<br>-HeLa cells were a gift of Sara Barozzi (Imaging Facility, IFOM-IEO Campus, Milan, Italy) and were not further authenticated.
Authentication	None of the cell lines used were authenticated. The original commercial source of the HeLa cell line is unknown
Mycoplasma contamination	Cell lines were regularly tested for mycoplasma contamination and the test found to be negative
Commonly misidentified lines<br>(See ICLAC register)	We did not use any misidentified cell line

Plants

Seed stocks	Not applicable
Novel plant genotypes	Not applicable
Authentication	Not applicable
